# Clinical guideline SEOM: cancer of unknown primary site

**DOI:** 10.1007/s12094-014-1244-0

**Published:** 2014-11-13

**Authors:** R. Collado Martín, A. García Palomo, L. de la Cruz Merino, P. Borrega García, F. J. Barón Duarte

**Affiliations:** 1 Medical Oncology Department, Hospital San Pedro de Alcántara, Avda Pablo Naranjo S/N, 10003 Cáceres, Spain; 2 Palomo Medical Oncology Department, Hospital de León, León, Spain; 3 Medical Onocology Department, Hospital Virgen de Macarena, Sevilla, Spain; 4 Medical Oncology Department, C. H. Universitario de Santiago, Calle Choupana, 15702 Santiago de Compostela (La Coruña), Spain

**Keywords:** Cancer, Unknown primary site, Chemotherapy, Diagnosis and treatment

## Abstract

Cancer of unknown primary site is a histologically confirmed cancer which is manifested in advanced stage, with no identifiable primary site after the use of standard diagnostic procedures. Patients are initially placed into one of categories based upon the examination of the initial biopsy: adenocarcinoma, squamous cell carcinoma, neuroendocrine carcinoma and poorly differentiated carcinoma. Appropriate patient management requires an understanding of several clinicopathologic features that help to identify several subsets of patients with more responsive tumors.

## Introduction

Cancer of unknown primary site (CUP) is by definition a histologically confirmed cancer which is manifested in advanced stage, with no identifiable primary site after the use of standard diagnostic procedures, which must include the PET [1]. It seems to represent 2–10 % of all malignancies [2], although there is a high variability among the series due to the difficulty in defining CUP. In any case, this entity includes a wide variety of presentations in a general framework of a poor prognosis and where the effort in diagnosis should be aimed at identifying cases for which treatment brings real benefits to the patient [3].

## Staging and prognosis

As advanced disease, the prognosis is poor. However, we can distinguish two main groups:

- Poor prognosis and limited therapeutic options (80–85 % of the CUP), which have a negative impact on survival (mOS <12 months). Features of this subgroup are males, the diagnosis of adenocarcinoma or squamous, multiorgan involvement, the PS and LDH. In this group we can still find two subgroups according to the PS (0–1 vs. ≥2), LDH (normal vs. no) and extent of disease (oligo vs. multiple). The combination of these, allows the identification of potential candidates for systemic treatment (PS 0–1 + normal LDH and oligometastatic disease) [4].
- The second group (15–20 %) represents patients where the effort in the diagnosis is justified because the treatment can provide clear benefits. We will refer mainly to this group.

## Diagnosis

Three rules should be respected to make an appropriate and efficient diagnosis of CUP:

1. The information from clinical, laboratory and radiological tests, should always guide the actions of the pathologist.
2. A suitable sample for study should be provided to the pathologist.
3. The pathologist must include in the study the conventional microscope and an immunohistochemical (IMH) algorithm. In some situations, this can be supplemented by other techniques (molecular profiles, electron microscope, genetic abnormalities).

The initial evaluation by conventional microscope must allow classification of CUP into several subtypes (first step):

**Table:** 

1st step: Conventional microscope			
Well and poorly differentiated adenocarcinomas	Undifferentiated carcinoma	Squamous cell carcinoma	Undifferentiated neoplasms
50 %	30 %	15 %	5 %

The next step is the IMH which is technique based on the use of antibodies against specific components of the cell. Some of these tests are broad spectrum and others are more specific. Among the first group, we distinguish those that detect membrane antigens, frontline IMH, which are applied only to undifferentiated forms (adenocarcinomas and poorly differentiated carcinomas and undifferentiated neoplasias). They allow to extract from this group lymphomas, sarcomas and melanomas (2nd step); beside them we find the cytokeratins, basically CK7 and CK20; they are used to classify carcinomas and adenocarcinomas (3rd step). Additional markers are often added to focus even more diagnosis (step 4).

**Table:** 

2nd step: IMH to membrane antigens			
Lymphoma	Melanoma	Carcinoma	Sarcoma
LCA (CD45); vimentin	S-100; HBM45; vimentin	EMA panCK AE 1/3 y CAM5.2	Vimentin

**Table:** 

3rd step: Cytokeratins CK 20 y 7		
	CK 20+	CK 20−
CK7+		Non-small cell lung cancer
		Breast
	Gastric	Serous ovarian
	Mucinous ovarian	Endometrial
	Pancreas	Pancreas
	Biliary tract	Biliary tract
	Urothelial origin	Mesothelioma
		Endocrine
		Germinal
CK7−	Colorectal	Squamous lineage
	Merkel cell	Prostate
	Gastric	Kidney
	Mucinous ovarian	Hepatocellular carcinoma

**Table:** 

4th step: IMH to carcinomas and specific adenocarcinomas	
Tumor	Specific staining
Urothelial origin	p63, thrombomodulin, uroplakin
Mucinous ovarian	WT-1
Serous ovarian	WT-1, BerEp4; Ca 125; RE; mesothelin
Lung subtype adenocarcinoma	TTF-1; Surf-A y B
Lung no adenocarcinoma	CK-7
Small cell lung cancer	Cromogranin A, sinaptofisine
Breast	RE; RPg; HER.2; mamoglobin; GCDFP15
Endometrial	RE; vimentine
Mesothelioma	WT-1; calretinin; mesothelin; CK5
Germinal tumors	Falc; AFP; βHCG; CD30; OCT4
Thyroid	Thyroglobulin; TTF-1
Prostate	PSA; falc
No urothelial kidney	CD10; vimentine; gp200
Colorectal	CX2; CEA
Hepatocellular carcinoma	Herpar1; CD10; pCEA
Pancreas/biliary tract	CDX2; CK7; Ca 19.9; mesothelin; trifoil factor 1
Neuroendocrine	Cromogranin A; sinaptofisine; CD56; PGP9

After applying all these steps you may still remain less than 5 % of “undifferentiated neoplasms”. Additional studies, including electron microscopy and some chromosomal test, will play a central role in this last group (Step 5).

**Table:** 

5th step: Electron microscopy and chromosomal test	
Findings	Origin suggestion
Premelanosomes	Melanoma
Secretory granules	Neuroendocrine tumor
IG gene rearrangements	Lymphoma B
Translocation t(11;22)(q24;q12)	PNET and Ewing sarcoma
Isochromosome 12 (i12p)	Extragonadal germ cell tumors
Translocation NUT (gen BRD4)	NUT midline
VEB	Cervical lymphadenopathy (nasopharynx)

Progress in microarray technology has allowed analysis of genetic signatures of the main tumors. It is possible to have platforms to identify the primary site, since the metastatic tissue retains these genetic firms partly. Some of these technologies are currently commercially available, with a diagnostic accuracy that reaches 80 %, but their use is very restricted at the present time and there are no prospective studies to compare them with the techniques of microscopy and conventional IMH.

## Clinical–diagnostic evaluation

All patients with conditions for a possible treatment should be routinely subjected to a battery of diagnostic tests to guide and assist the pathologist in his work. We will divide these into those *sine qua non* a metastatic lesion should not be defined as CUP, pre-pathological study, and others to be specific depending on the findings of these and the profiles IMH reported by the pathologist.

Between the first and still with no broad consensus, there shall be listed:

1. Detailed medical history, including a history of other comorbid conditions, neoplasms, interventions, etc., and family history, to be followed by a complete physical examination, including breast exam, anorectal and gynecological.
2. Basic laboratory test: blood count, kidney and liver function, electrolytes, calcium and urinalysis.
3. Fecal occult blood.
4. Chest–abdominal–pelvic CT.
5. Endoscopic tests based on symptoms–signs guide.

The inclusion of FDG–PET in the routine test has controversies [5], except for patients with suspected pulmonary primary tumors or squamous-cell cervical lymphadenopathy due to the lack of prospective studies.

Other techniques will be necessary only if the clinical situation and the pathologist’s report guide into subtypes where a bigger diagnostic effort is worthwhile. These situations can be summarized as follows:

1. Carcinomas and poorly differentiated adenocarcinomas in young patients with predominant involvement of the midline and IMH suspected germ cell tumor. The serum AFP and βHCG and testicular ultrasound and also the detection of isochromosome i12p can help the diagnosis.
2. Women with peritoneal carcinomatosis and histologies with papillary configuration and/or Psammoma bodies whose IMH suggest ovarian cancer or family history suggestive of breast–ovarian syndrome. Serum determination of marker CA 125, invasive pelvic examination and even diagnostic–therapeutic laparotomy may be indicated.
3. Women with axillary metastases of adenocarcinoma with IMH suspected breast cancer (especially mammaglobin, CK7/CK20 and GCDFP-15; ER and PR can be negative). Ultrasound/mammography should be practiced and even MRI of both breasts. The use of marker Ca 15.3 can be used as support. However, the absence of radiological data does not rule out a breast primary (“occult breast carcinoma”).
4. Male with predominantly blastic bone metastases. Although the IMH can be of great value, this clinical situation always advised PSA screening and detailed urological examination.
5. Patients with adenocarcinomas presenting with liver metastases and/or peritoneal carcinomatosis, and IMH show “colorectal profile”. The therapeutic development in colorectal cancer forces to discard it as primary; gastrointestinal endoscopy is recommended, even in the absence of positive fecal occult blood or symptoms–signs guide.
6. Patients with adenocarcinomas and one metastatic site. Although in most cases, the existence of other foci is evident in the evolution, the radical treatment of the shown lesion brings benefits in some patients. PET is indicated.
7. Cervical or inguinal lymphadenopathy with squamous histology in patients with good PS. In the first case, the endoscopic exploration of ENT and pulmonary area are indicated, and also the PET, as quoted above. In the case of inguinal involvement it is indicated for anorectal, urological and gynecological inspection.
8. Tumors with neuroendocrine differentiation. Although they are often initially recognized due to their consistent histologic features and related IMH, it is sometimes necessary to include nuclear medicine techniques such as OctreoScan, given that 80 % of these lesions have high levels of somatostatin receptors [6]. Other invasive tests can be added, such as capsule endoscopes and endoscopic ultrasound. For patients with carcinoid syndrome, detecting urinary 5-HIAA is highly specific for serotonin-producing tumors. The pathological study must include as required information, the mitotic index and the percentage of cells expressing Ki-67 or MIB.1, which is essential for classification in low, intermediate or high grade.

## Treatment: adenocarcinoma of unknown primary site

Adenocarcinoma of unknown primary site comprises approximately 60 % of cancer of unknown primary site. If a primary site can be identified, treatment is based upon the usual treatment for advanced cancer arising from that site. But if this is not possible, until the 40 % of patients with adenocarcinoma of unknown primary site will contain several clinically defined subgroups for which specific therapy will be available (IV, B):

- Women with peritoneal carcinomatosis from papillary carcinoma: surgical cytoreduction should be considered followed by chemotherapy regimens that are effective in the treatment of advanced ovarian cancer.
- Women with localized adenocarcinoma involving axillary nodes: these patients are treated according to guidelines for stage II–III breast cancer, with axillary node dissection and modified radical mastectomy or radiotherapy.
- Men with skeletal metastases: particularly if the metastases are blastic and patients have a significantly elevated serum PSA. Even when clinical features do not suggest prostate cancer, is reason for trial of hormonal therapy with bisphosphonates.
- Patients with a colon cancer profile (adenocarcinoma with histology typical of gastrointestinal origin, predominant metastatic sites and liver and/or peritoneum, typical immunohistochemical staining pattern including CK20-positive/CK7-negative or CDX-2 positive): patients with this profile respond well to chemotherapy with contemporary regimens developed for patients with metastatic colorectal carcinoma.
- Localized adenocarcinoma occurring in the mediastinum: most likely from either a germ cell tumor or a non-small cell lung cancer. Patients younger than 40 years should be treated for poor-risk germ cell tumors. Patients aged 50 years or older should be treated according regimens for patients with non-small cell lung cancer. Those between 40 and 50 years of age should be treated with empiric, platinum-based regimens in the absence of additional diagnostic information.

### Chemotherapy

Empiric chemotherapy remains the treatment of choice for the small minority of patients with adenocarcinoma of unknown primary site who do not fit into any of the clinical subgroups outlined above or those patients in whom a tissue of origin cannot be predicted after a complete diagnostic evaluation that includes molecular tumor profiling.

No specific can be recommended as standard of care [7, 8]; therefore some authors suggest that chemotherapy be limited to symptomatic patients with PS 1 to 2 or to asymptomatic patients with a PS of 0 and aggressive cancer [9]. Therefore, participation in clinical trials should be strongly encouraged, but if this is not possible, we recommend regimens which have shown efficacy in phase II and III studies:

- Paclitaxel and carboplatin with or without etoposide: the triple drug regimen shows comparable efficacy as compared to gemcitabine/irinotecan [10], but reported significantly less toxicity with the two-drug regimen and equal survival rates, with median survivals of 7–10 months (I, A). So, several two-drug combinations are reasonable choices for first line therapy.
- Carboplatin with docetaxel: docetaxel in combination with either cisplatin or carboplatin was active in patients with adenocarcinoma and poorly differentiated carcinoma and was better tolerated with carboplatin, with a median survival of 8 months.
- Cisplatin with gemcitabine: this combination was found to be better than that of the cisplatin and irinotecan regimen (I, A) with median survival rates of 8 months [11].
- Gemcitabine with docetaxel: this regimen was found to be well tolerated with a median survival of 10 months.
- Capecitabine or 5-Fu with oxaplatin [12]: the combination of capecitabine and oxaliplatin appears to be active and well tolerated for this patient population with median survival 9.7 months (IV, B).

### Targeted therapies

Whether targeted agents should be used or not in these kind of patients is still unknown. It has been reported in a phase II trial that the combination of bevacizumab and erlotinib (alone or combined with paclitaxel and carboplatin) has substantial activity as first or second line therapy with 27 % overall survival at 24 months.

### Surgery

Surgery can also be considered, apart from axillary node resection and surgical cytoreduction in peritoneal carcinomatosis, in:

- Lung nodules: surgery can be considered for respectable lung nodules, and chemotherapy can be considered with or without resection.
- Inguinal nodes: lymph node dissection is recommended for inguinal nodal involvement. Radiotherapy with or without chemotherapy can also be indicated if clinically indicated (II, B in the case of bilateral inguinal node involvement, for the use of radiation therapy).
- Liver lesions: surgical resection with or without chemotherapy is recommended for patients with localized adenocarcinoma in the liver. Other locoregional therapeutic options can include chemoembolization, radiofrequency ablation or percutaneous ethanol injections.
- Bone lesions with potential for fracture: surgery and/or radiation therapy may be recommended.
- Brain metastases: evidence suggested survival benefits from tumor resection for selected patients of good prognosis with up to three metastatic sites.

To summarize, if no other site of disease involvement can be identified, we recommend definitive local therapy, consisting of either surgical resection or radiation therapy.

### Radiation therapy

Radiotherapy is a treatment option for a variety of localized tumors, particularly as follow up treatment after other locoregional therapeutic options. Radiation therapy alone may also be considered for:

- Bone lesions
- Retroperitoneal mass with a non-germ cell histology (II, B)

Final recommendation: Empiric chemotherapy remains the treatment of choice for patients in whom molecular profiling fails to predict a tissue of origin. We recommend that patients should be enrolled in formal clinical studies whenever possible.

Chemotherapy regimens for adenocarcinoma of unknown primary sites.

**Table:** 

Chemotherapy (mg/m^2^)	Time	Interval
Paclitaxel 175	Day 1	3 weeks
Carboplatin 5 AUC	Day 1	
Docetaxel 75	Day 1	3 weeks
Carboplatin 5 AUC	Day 1	
Cisplatin 60–75	Day 1	3 weeks
Gemcitabine 1000	Day 1 + 8	
Gemcitabine 1000	Day 1 + 8	3 weeks
Docetaxel 75	Day 1	
Oxaliplatin 130	Day 1	3 weeks
Capecitabine 2000	Day 1−14	
Gemcitabine 1000	Day 1 + 8	3 weeks
Irinotecan 100	Day 1 + 8	

## Treatment: squamous cell carcinoma of unknown primary site

Squamous cell carcinomas comprise approximately 5 % of cancers of unknown primary site [13]. Effective treatment is available for some patients who fit certain clinical syndromes:

- Squamous carcinoma involving cervical lymph nodes: these patients should be treated according to the recommendations for treatment of primary head and neck cancers (II, B). Metastatic disease in neck lymph nodes only, particularly in the upper and middle cervical nodes, is potentially curable with radiotherapy or node dissection under appropriate circumstances. For advanced stages, induction chemotherapy with platinum-based combination or chemoradiation is also reasonable [14].
- Squamous carcinoma involving inguinal lymph nodes: lymphadenectomy with or without postoperative radiation therapy to the inguinal area, sometimes results in long term survival (II, B). Chemotherapy can also be considered for this group of patients.
- Squamous carcinoma metastatic to other sites: Patients with site-specific squamous carcinoma in the mediastinum, lower cervical or supraclavicular lymph nodes, should be treated according to the guidelines for non-small lung cancer. Other rare presentations include primaries from esophagus, uterine cervix, anus and skin.

### Chemotherapy

According to the recommendations for treatment of adenocarcinoma of unknown primary site, in those patients with disseminated squamous cell carcinoma of unknown primary, a trial of therapy is preferred, with the additional recommendations of symptoms control and the consideration of empiric systemic chemotherapy (II,C), especially, in patients with good performance status [15].

- Paclitaxel and carboplatin: in the Hellenic Cooperative Oncology Group phase II, one patient had an objective response of 3 months duration after paclitaxel and carboplatin.
- Docetaxel and carboplatin: this combination was assessed in a phase II trial, with a response rate 32 % and median OS of 16.2 months.
- Paclitaxel with cisplatin: In a phase II study of patients with unfavorable presentations, 3 of the 31 patient had SCC. The regimen gave an overall response rate of 42 %, and the median OS was 11 months
- Docetaxel with cisplatin: the safety and efficacy of this regimen has been assessed in 45 patients with occult primary tumors. The reported overall response rate was 65.1 %, and the median OS was 11.8 months.
- Cisplatin and 5-Fu: Kusaba et al. reported a response ratio of 54.5 % and a median OS of 10 months.
- Cisplatin with docetaxel and 5-Fu: in a randomized phase III trial with chemotherapy followed by chemoradiation, the overall response rates after induction chemotherapy were 72 %.
- Cisplatin with gemcitabine: The GEFCAPI02 trial compared cisplatin to cisplatin and gemcitabine. There was a trend towards better OS with the addition of gemcitabine.
- mFolfox6: this regimen is used in squamous cell cancer of the esophagus and stomach and could be useful in other squamous cell cancers of unknown primary.

### Surgery and radiation therapy

Surgery and/or radiation therapy, in order to save fracture, are options for patients with an isolated bone lesion and good performance status. On the other side, patients with limited remove metastasis should be managed with surgical resection followed by whole brain radiation therapy or stereotactic radiosurgery. And as we said previously, radiotherapy may be also recommended for supraclavicular nodal involvement in site-specific squamous cell cancer or after lymph node dissection for the involvement of axillary or inguinal nodes if more than two nodes are involved or extracapsular extension is presented.

Final recommendation: Empiric chemotherapy remains the treatment of choice for patients in whom molecular profiling fails to predict a tissue of origin. We recommend that patients should be enrolled in formal clinical studies whenever possible.

Chemotherapy regimens for squamous cell carcinoma of unknown primary sites.

**Table:** 

Chemotherapy (mg/m^2^)	Time	Interval
Paclitaxel 175	Day 1	3 weeks
Carboplatin 5 AUC	Day 1	
Docetaxel 75	Day 1	3 weeks
Carboplatin 5 AUC	Day 1	
Paclitaxel 175	Day 1	3 weeks
Cisplatin 60	Day 1	
Docetaxel 60	Day 1	3 weeks
Cisplatin 80	Day 1	
Cisplatin 60–75	Day 1	3 weeks
Gemcitabine 1000	Day 1 + 8	
mFolfox6		2 weeks
Oxaliplatin 85	Day 1	
Leucovorin 400	Day 1	
5-Fu 400	Day 1 bolus	
5-Fu 1200	×2 Days (total 2,400 over 48 h) continuous infusion	
Docetaxel 75	Day 1	3 weeks
Cisplatin 75	Day 1	
5-Fu 750	Days 1–5 continuous infusion	
Cisplatin 20	Day 1–5	4 weeks
5-Fu 700	Day 1–5 continuous infusion over 24 h daily	

## Treatment: poorly differentiated cancer from an unknown primary site

When specialized pathological studies identify treatable tumor types like poorly differentiated lymphoma, extragonadal germ cell tumor, melanoma or sarcoma, treatment should be based upon that diagnostic category.

For those patients whose evaluation is consistent with a poorly differentiated carcinoma, empiric, platinum-based regimens are recommended (see “adenocarcinoma of unknown primary site”) [16, 17].

Final recommendation: Empiric chemotherapy remains the treatment of choice for patients in whom molecular profiling fails to predict a tissue of origin. We recommend that patients should be enrolled in formal clinical studies whenever possible.

### Levels of evidence

Levels of evidence (from I to IV) and grades of recommendation (from A to D) are given in square brackets, as used by the American Society of Clinical Oncology. Unless otherwise noted, all recommendations are considered justified, standard clinical practice and apply to most patients unless a clear and compelling rationale for an alternative approach is present.
